# Hypoxia: A key feature of COVID-19 launching activation of HIF-1 and cytokine storm

**DOI:** 10.1186/s12950-020-00263-3

**Published:** 2020-10-29

**Authors:** Mozhgan Jahani, Sadat Dokaneheifard, Kamran Mansouri

**Affiliations:** 1 Medical Biology Research Center, Health Technology Institute, Kermanshah University of Medical Sciences, Kermanshah, Iran; 2 Department of Human Genetics, Sylvester Comprehensive Cancer Center, University of Miami Miller School of Medicine, Miami, Florida 33136 USA; 3 Department of Molecular Medicine, School of Medicine, Kermanshah University of Medical Sciences, Kermanshah, Iran

**Keywords:** COVID-19, Hypoxia, HIF-1α, Cytokine storm

## Abstract

COVID-19, disease caused by the new coronavirus, SARS-CoV-2, appeared in the end of 2019 and was rapidly spread in most countries. This respiratory virus has different symptoms from moderate to severe, and results in lung pneumonia following acute respiratory distress syndrome (ARDS) and patient’s death in severe cases. ARDS is a severe form of acute lung injury that is caused by high inflammatory response of the innate immunity cells. Hypoxia is the common feature in the inflammatory sites with having various impacts on this condition by induction of some factors such as hypoxia inducible factor-1α (HIF-1α). HIF-1α regulates some important cellular processes including cell proliferation, metabolism and angiogenesis. Furthermore, this factor is activated during the immune responses and plays important roles in the inflammation site by inducing pro-inflammatory cytokines production through immune cells. So, in this study the possible effect of the HIF-1α on the COVID-19 pathogenesis with emphasizes on its role on innate immunity response has been discussed.

## Introduction

Coronaviruses (CoV) are a large family of viruses causing illness ranging from the common cold to more severe diseases. At the end of the 2019, prevalence of the new coronavirus, SARS-CoV-2, was reported in Wuhan, Hubei, China which its related disease was called “the Corona Virus Disease 2019; COVID-19”. In addition to china, COVID-19 was rapidly spread in other countries. COVID-19 has high rate of transmission and its symptoms are moderately acute in patients [1]. SARS-CoV-2 infection causes respiratory illness as its commonly symptoms are fever, cough or sneeze which can also cause pneumonia, acute respiratory distress syndrome (ARDS), respiratory failure, shock, as well as organ failure and patients death in severe cases [1, 2].

Additionally, there are a number of patients at higher risk for COVID-19 infection, including patients with cancer, transplants, or other conditions [3]. In this case, people with a lung cancer and smoking/vaping history may be remain an important vulnerable population [4, 5]. Lung cancer patients are very susceptible for getting respiratory infections and most of them are detected with chronic obstructive pulmonary disease (COPD) as well as metastatic disease, so it is expected to observe greater mortality or at least greater severity of the infectious symptoms. In a study conducted by Jacobo Rogado et al., increased mortality of patient with active lung cancer or were on active treatment was detected [5].

In relation to smoking/vaping people, various indirect studies demonstrated that this population is at a higher risk to show severe symptoms and need mechanical ventilation as compared to non-smokers [4]. Some previous studies indicated number of reasons for this complication in smoking patients. Growing body of evidences has been reported that SARS-CoV-2 infects lung cells using human angiotensin-converting enzyme 2 (ACE-2) receptors. ACE-2 is an integral glycoprotein in the cell membrane expressed in the various cells including epithelial cells in lungs, kidney, intestine and blood vessels. So, it can have adverse effects on these organs function. ACE-2 catalyzes angiotensin II conversion to angiotensin 1–7 in renin angiotensin system. Angiotensins 1–7 are vasodilator and have protective effects on the cardiovascular system [6]. Interestingly, ACE-2 expression is upregulated in epithelial cells of airway in smoker therefore smoking patients can be associated with pathologies like COPD and idiopathic *pulmonary fibrosis* (IPF) [7, 8]. Increasing the cathepsin B expression, oxidative stress and inflammatory responses in the lung of the smokers/vapers which increases the membrane permeability and susceptibility towards viral/bacterial infections are the other reasons for smoker susceptibility to COVID-19 infection. It has interestingly been shown that the expression of IL-6, TNF-α and other pro-inflammatory cytokines are increased in chronic smoking condition [9].

Furthermore, it has been suggested that SARS-CoV-2 recruits the host cells autophagy pathway components for replication [10]. So, using the anti-inflammatory drugs, some antiviral drugs and autophagy inhibitors [11, 12] as well as some renin-angiotensin enzymes inhibitors [13–15] are the common therapies for patients with COVID-19. However, despite many efforts to produce the vaccine or effective drugs, there is no vaccine or drug specific to eradicate this pandemic disease yet. Innate immunity response, as the first line of defense against microorganisms, plays essential roles in prevention of viral infection and invasion. So that, many pro-inflammatory cytokines are produced after viral infection to eliminate viruses in the body by promoting of inflammation caused by *innate* and acquired *immune* cells involvement [16]. Corresponding to related studies of previous coronavirus infection, innate immune responses can be a protective or destructive responses, and it may be considered for immune intervention [17]. In COVID-19, acute tissue inflammation of lung, as the main organ of virus infection and proliferation, causes threatening condition for patient’s life [18]. So, in SARS-CoV-2 infection, innate immunity regulation is critical for decreasing the lung injury and increasing the patient survival rate due to hyper-inflammation effect of cytokines produced by inflammatory cells (neutrophils and macrophages) in the lung.

As a dominant micro-environmental property of innate immunological activity, hypoxia occurs at the inflammation sites [19, 20]. So, hypoxia exists in severe pneumonia and respiratory distress following SARS-CoV-2 infection [21]. HIF-1α is a critical factor that is activated in hypoxic conditions. It has pro-inflammatory effect via regulation of high level of IFNI which is produced by cytokines production, such as Interleukin 6 (IL-6) and tumor necrosis factor alpha (TNF-α) as well as activation of the signal transducer and activator of transcription 3 (STAT3) pathway to take in the inflammatory process [22].

So that, at this micro-environmental condition with hypoxia and HIF-1 activity, suppression of HIF-1 transcription or inhibition of its activity can be effective in reducing the inflammation caused by viral infection in involved organs such as lung in COVID-19. Therefore, in this review we have summarized the innate immunity effects in viral infection with emphasis on its impact in SARS-CoV-2 infection. Furthermore, HIF-1, as one of the critical factors in inflammation process which its activity inhibition potentially affects the controlling of COVID-19, has been discussed.

### Innate immunity against viral infection

**Fig. 1:**
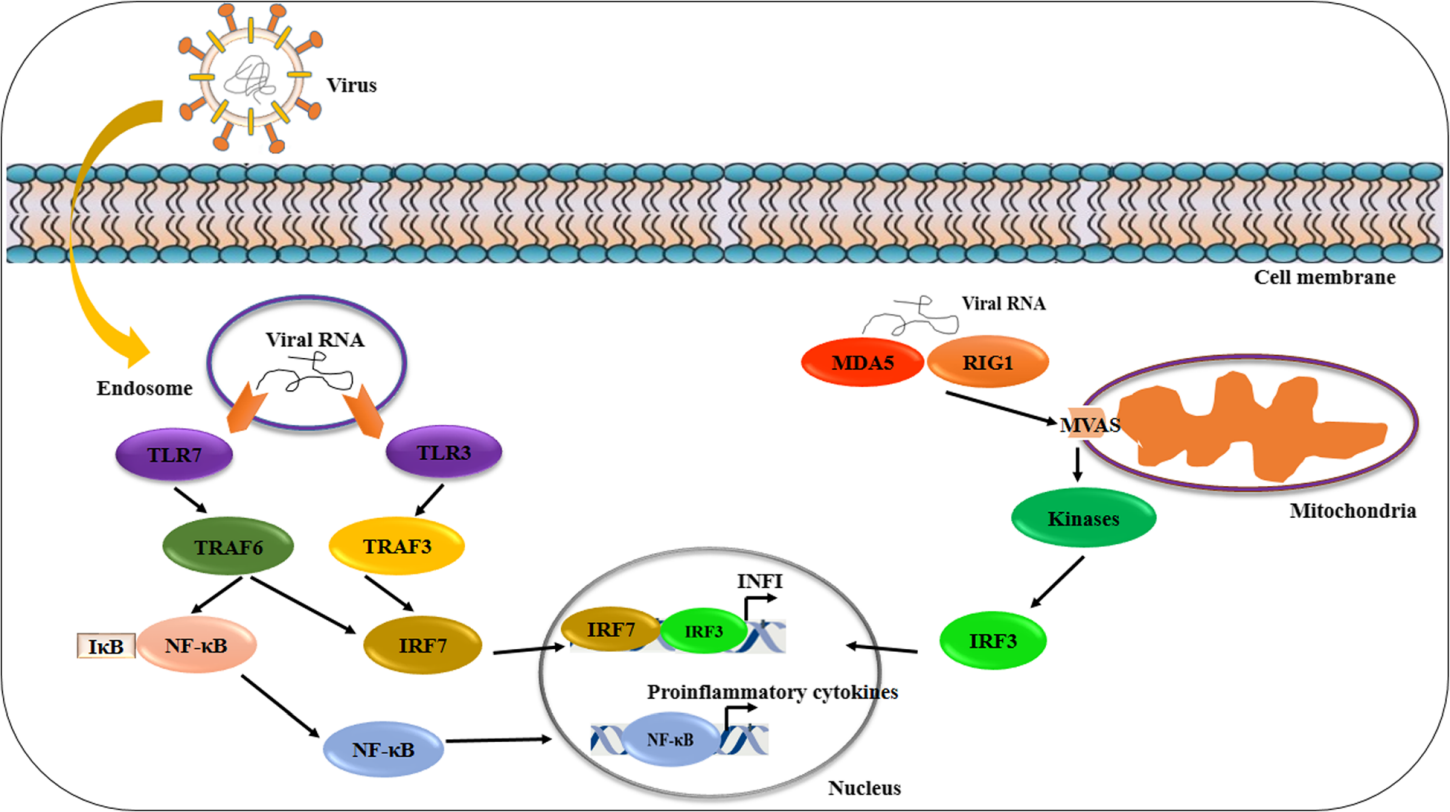
Innate immune response to RNA viruses. Innate immune response is activated after viral PAMPs (such as viral RNA) detection by host cells PRR including TLRs (TLR3, TLR7) and RLRs (MDA5 and RIG1). Thereafter, PRR interaction with mitochondrial antiviral-signaling protein (MAVS) activates several kinases. Furthermore, some adaptor proteins including tumor necrosis factor receptor (TNFR)-associated factor 6 (TRAF3) and TRAF6 are activated by TLRs. Both kinases and adaptor proteins activate IRF3 and IRF7 transcription factors as well as NF-κB transcription factor. IRF3 and IRF7 nuclear translocation result in INFI expression. Proinflammatory cytokines are induced after NF-κB translocation into the nucleus

Innate immunity is stimulated by viruses giving rise to the antiviral responses in host cells. Innate immunity is triggered by recognition of pathogen-associated molecular patterns (PAMPs) through different pattern recognition receptors (PRRs), including Toll like receptors (TLRs), retinoic acid inducible gene-I- (RIG-I-) like receptors (RLRs) and NOD-like receptors (NLRs) [23]. Various viral components including genomic DNA, single-stranded RNA (ssRNA), double-stranded RNA (dsRNA), RNA with 5′-triphosphate ends and viral proteins are recognized by host PRR [24]. Regarding to RNA viruses such as SARS-CoV-2, PAMPs are viral genomic RNAs and also dsRNAs produced in replicated viruses which are recognized by TLR3, TLR7, the cytosolic RNA sensor, and RIG-I/MDA5. Following virus component binding to TLR and RLR receptors, some downstream signaling pathways including nuclear factor-κB (NF-κB) and *interferon regulatory factor 3 (*IRF3) are activated and then translocated into the nuclear space. In the nucleus, these transcription factors not only induce the *interferon I* (IFNI) expression as well as other pro-inflammatory cytokines and chemokines but also increase the expression of CD40, CD80 and CD86 co-stimulatory molecules [25] (Fig. 1).

IFNI, the main factor in antiviral response of innate immunity, binding to its receptor triggers the Janus kinase-signal transducers and activators of transcription (JAK-STAT) pathway and regulates the expression of some important genes, such as protein kinase R (PKR), which are involved in viral component elimination from infected cells. Furthermore, apoptosis induction in the infected cells and resistance of uninfected cells to the virus infection occurs following INFI binding to its receptor. On the other hand, pro-inflammatory cytokines and also chemokines are essential for virus elimination by promoting inflammation and recruiting innate and acquired immune cells [25].

### HIF-1 structure and stability

HIF-1 is a conserved heterodimer transcription factor regulated through O2 concentration and availability. This transcription factor includes two subunits, an O_2_-regulated HIF-1α and consistently expressed HIF-1β which both of them are related to basic helix-loop-helix (*bHLH*) and Per-Arnt-Sim (PAS) domain protein family [26–28]. Although HIF-1β is permanently expressed in the cell, the HIF-1α expression rises progressively in hypoxic condition. HIF-1α dimerization with HIF-1β in the nucleus [28] and subsequent binding of this complex to hypoxia-response elements (HREs) in the target genes results in transcription of genes which are essential for an adaptive responses against hypoxia including glycolytic enzymes, glucose transporters, erythropoietin and angiogenic factor vascular endothelial growth factor (VEGF) [29].

HIF-1α gene is comprised of 15 exons and 14 introns [30]. Proteins encoded by this gene possess a bHLH and PAS domain being necessary for its dimerization with HIF-1β and DNA binding. There are two transactivation domains (TAD) in c-terminal of HIF-1α named N- terminal TAD (N-TAD) and C-terminal TAD (C-TAD) which are separated by an inhibitory domain, and involved in transactivation through directly or indirectly interaction with co-activators including P300/ CREB-binding protein (P300/CBP) and Ref as components of the transcription initiation complex [31]. Tight adjustment of HIF-1α stability and its transactivation function is controlled by posttranslational transcription alterations such as hydroxylation, ubiquitination, acetylation, and phosphorylation. These alterations usually occur in multiple domains of this protein [32]. In normoxic conditions, ubiquitination of two proline and one lysine residues in oxygen dependent degradation domain (ODDD) of HIF-1α causes its ubiquitination and its further degradation [33]. Furthermore, HIF-1α hydroxylation, catalyzed by prolyl-hydroxylase domain proteins (PHDs) and O2 as a substrate, is needed for its degradation in normoxic condition. There are four PHD isoforms, however only PHDs 1–3 are involved in HIF-1α hydroxylation. Nonetheless, in hypoxic conditions cell survival is dependent on preserving of this protein and crying down these processes.

### HIF-1 roles in viral infection and innate immunity

Viral infection can induce HIF-1α activation which the net consequence of its activation can give favors the pathogen rather than the host [34]. Different types of viruses have used various mechanisms stabilizing HIF-1α with an anti-apoptotic effect on the infected cells.

For example it has been demonstrated that HIF-1α is stabilized by Hepatitis C virus (HCV) protein [35]. Furthermore, the upregulateion of the HIF-1α-controlled genes expression including those coding for glycolytic enzymes was observed in HCV infected cells. It has been shown that long-term expression of HCV protein decreases the mitochondrial oxidative phosphorylation, and higher use of glycolysis pathway preserves the cell survival. It seems that HIF-1α stabilization and glycolytic enzymes upregulation mediate this adaptive response to mitochondrial damage induction by HCV [35]. Increasing the activity of HIF-1α by Hepatitis B-encoded X protein (HBx) protein has been exerted through reinforcement of this transcription factor assembly with cAMP-response element binding protein (CREB)-binding protein (BP) [36].

HIF-1α protein expression level is also increased via degradation of PHD1 and PHD3 by Epstein- Barr virus (EBV) oncoprotein latent membrane protein (LMP1) [37]. Influenza viruses stabilize HIF-1α through impairing proteasome function and decreasing the expression of factor inhibiting HIF-1α (FIH-1) [38]. The high production of pro-inflammatory cytokines, cytokine storm, as a key contributor to severe pneumonia in patients with H1N1 infection is mediated by HIF-1α which can induce proinflammatory molecules production in the site of inflammation [39].

Human papillomaviruses (HPVs) are involved in various types of malignancies; more than 99% of cervical cancers. A former study has indicated that HIF-1α protein level is increased in hypoxia when HPV oncogenes are present, as this was true for all types of risk viruses [40]. Furthermore, it has been shown that HPV-16 E6 and E7 oncoproteins can result in non-small cell lung cancer (NSCLC) progression likely by increasing the tumor angiogenesis via HIF-1alpha/VEGF pathways which may be considered as a potential molecular targets for HPV-related NSCLC44 treatment [41].

So, due to viral effect on the innate immunity reaction and because of HIF-1α effect on the promotion of viral infection, understanding the role of HIF-1α on innate immunity response can be effective for introducing new strategies against viral infection.

HIF-1α is expressed in most cell types such as immune cells, and regulates some cellular functions including cell metabolism and inflammation [42–44]. HIF-1α expression is induced in response to hypoxia condition at the site of inflammation. Phagocytic cells including macrophages and neutrophils are present in the infected tissues with hypoxic microenvironments and have critical roles in innate immune response against pathogens such as viruses [16]. They express low level of HIF-1α under normal pressure of oxygen in blood stream. However, when they encounter with low oxygen pressure in the site of infection they increase HIF-1α expression promoting their phagocytic activity [22]. Furthermore, HIF-1α transcription activity increases phagocytes cells survival and stimulates the expression of some important factors including VEGF as well as pro-inflammatory cytokines (TNF, IL-1 and IL-12) in the site of infection [45].

All cell types need energy (in the form of ATP) to perform their critical functions in the body. Two cellular metabolic pathways which are used for ATP production from glucose are glycolysis and tricarboxylic acid cycle (TCA) [46]. Metabolic pathways are flexible, and metabolic changes can occur in response to availability of nutrients as well as oxygen levels. Glycolytic pathway is activated under hypoxic condition due to low oxygen level for promotion of oxidative phosphorylation (OXPHOS) process [47]. As an important example, the metabolic changes toward glycolysis (Warburg effect) are critical processes in cancer cells because of their high energy demand for proliferation especially in hypoxic condition. Metabolic changes are controlled and regulated by different factors that among them HIF-1α is critical during hypoxic condition [48]. HIF-1α induces some glycolytic enzymes including hexokinase and phosphofructokinase as well as glucose transferases (GLUTs) at the cell surface [47] as HIF-1α inhibition has been one of the important strategies in cancer therapy [49–51].

Immune cells have different energy requirements according to their activation state, and they must be able to alter their metabolism. There is no metabolic alteration in quiescent immune cells but when they are activated they need metabolic changes for providing their energy demand [46, 52]. As mentioned above, HIF-1α, as a decisive factor in immunity and inflammation regulation, can also provide this metabolic switch in immune cells [46].

Glycolysis pathway is the prominent source of energy production in neutrophils as well as Dendritic cells (DCs) and macrophages [53]. Stimulation of metabolic reprogramming in immune cells such as neutrophils has been described and the shift to glycolytic phenotype (the Warburg –like shift) was first reported in neutrophils in 1959 [54]. Glucose uptake enhancing and O2 consumption by neutrophils are regulated by HIF-1α signaling. Furthermore, HIF-1α extends neutrophil’s lifespan through inhibition of apoptotic signaling and also increasing their antimicrobial function by up-regulation of involved molecules [55].

HIF-1α regulates the expression of some metabolic intermediates such as GLUT-1 due to their HRE site. So that, glycolysis inhibition using the glucose analogue 2-deoxyglucose (2-DG) has shown the inhibition of DCs maturation in the pathway which HIF-1α is involved [43]. HIF-1α can also regulate macrophage functions by impact on its metabolism. It has been shown that HIF-1α is stabilized by M1 signals. On the other hand, HIF-1α activity is crucial for macrophages functions and polarization. HIF-1α gives favors the pro-inflammatory M1 macrophage polarization as its transcription activity is needed for macrophages metabolic switching to glycolysis through induction of glycolytic gene expression and glucose transporters in response to inflammatory stimuli [56]. M1 associated genes such as those encoding TNF-α, IL-1β, inducible nitric oxide synthase (INOS) and IL-23 are also increased in response to HIF-1α stabilization and its association with some metabolic intermediates (such as pyruvate kinase isozymes M2 (PKM2) and pyruvate dehydrogenase kinase 1 (PDK1) in the nucleus [22, 57].

Taken together, innate immunity response to viral infection and its related hypoxic microenvironment is highly dependent on the expression and activity of HIF-1α. This transcription factor promotes inflammation via up-regulation of HRE containing genes in pro-inflammatory immune cells including neutrophils, DCs and macrophages.

Inappropriate responses result in tissue destruction, vascular damage and organ failure, although the proper inflammation helps to eradication of infectious agents and maintenance of tissue integrity. Therefore, considering the HIF-1α effect on inflammatory response to pathogens and its improper activity can exacerbate the inflammation and leading to tissue damage.

### COVID-19 and innate immunity

It has been reported that COVID-19 cases (about 80%) are asymptomatic or have mild symptoms; however some of them have severe or critical condition which may lead to their death. It seems that the COVID-19 severity and mortality rate are more moderate than other CoV, severe acute respiratory syndrome (SARS) and *Middle East Respiratory Syndrome* (MERS). The most common symptoms of COVID-19 are fever, fatigue, and respiratory symptoms. COVID-19 disease severity and death is related to neutrophils proliferation elevation and lymphocytes population reduction (lymphopenia) in patients [2, 58]. As it is reported about host innate immune status of SARS-CoV-2 infected patients, there are increased amount of total neutrophils (38%) and c-reactive protein (84%) as well as high-levels of pro-inflammatory cytokines including IL-2, IL-7, IL-10, granulocyte-colony stimulating factor (G-CSF), interferon γ–induced protein-10 (IP-10), monocyte chemoattractant protein-1 (MCP-1), macrophage inflammatory proteins-1A (MIP-1A), and TNFα in patients with severe symptoms indicating the pro-inflammatory condition roles in disease progression and its severity [2, 59]. cytokine storm has been considered to have a critical role in COVID-19 pathogenesis. So that, lung injury induction by inflammation induced by “cytokine storm” can result in some implications such as pneumonia and ARDS leading to organ failure and patients death [2]. It is unknown whether SARS-CoV-2 can infect any immune cells or not. Since only small number of monocytes/macrophages can express SARS-CoV-2 receptor in the lung, other receptors for this virus or other way of virus cell entry such as antibody-dependent may exist [1, 60].

As previously mentioned, type I IFN and its downstream signaling cascade play critical roles in effective responses of innate immune against viral infection. The elevation of IFNI level can control, decrease the viral replication and induce adaptive immune response [25]. Regarding to SARS-CoV-2 modulation effect on the immune system, it can be considered that due to its genomic sequence similarity to SARS-CoV or MERS-CoV genomes, it may use similar strategy to interfere with host immune response such as prevention of the IFNI response in the early phase of infection. Accordingly, because of virus ability to transmit from asymptomatic patient, the fact is expected that SARS-CoV-2 can delay the early response of the innate immune by decreasing the IFNI expression [17]. Furthermore, it can be considered that innate immunity response is suppressed or ineffective in an early phase of virus infection. However, after viral proliferation and progression, high level of IFNI is produced, and phagocytes (neutrophils and macrophages) produce high amount of pro-inflammatory cytokines (cytokine storm) influx into tissue site of infection [17].

### Potential effects of HIF-1 on the COVID-19 related ARDS

Lung is a vital organ exposed to high amount of oxygen. This organ is sensitive to pathogen related infections including viruses, bacteria, and fungi. ARDS, a severe form of acute lung injury following hyper-inflammation, is one of the life-threatening symptoms of respiratory system viruses especially for SARS-CoV-2 virus [61]. In this syndrome, alveolar and interstitial edema occur due to increasing the permeability of the pulmonary capillary endothelial and alveolar epithelial barriers leading to infiltration of protein-rich fluid and immune cells into parenchyma [61]. Moreover, fluid accumulation in the alveoli decreases the effectiveness air exchange among the alveoli and vasculature which then results in hypoxemia and regional alveolar hypoxia [62].

Inflammation as well as hypoxia stimuli result in HIF-1α stabilization in alveolar epithelial cells. HIF-1α stabilization is also depends on succinate dehydrogenase induction followed by PHD activity inhibition in these cells [63]. It has been demonstrated that HIF-1α deletion in alveolar epithelial cells is associated with increased morbidity and mortality in stretch-induced acute lung injury (ALI). However, there are some different results in the case of viral infection [64]. So that, Xi et al. have reported that HIF-1α activation in alveolar epithelial cells caused by influenza virus results in dysfunctional alveolar remodeling. In addition, they have indicated that hypoxia dependent HIF1α activity is a determinant factor in epithelial cell fate, as mice epithelial cells without HIF1α expression have been recovered more rapidly with improved expansion of the type II alveolar cell population [65].

**Fig. 2:**
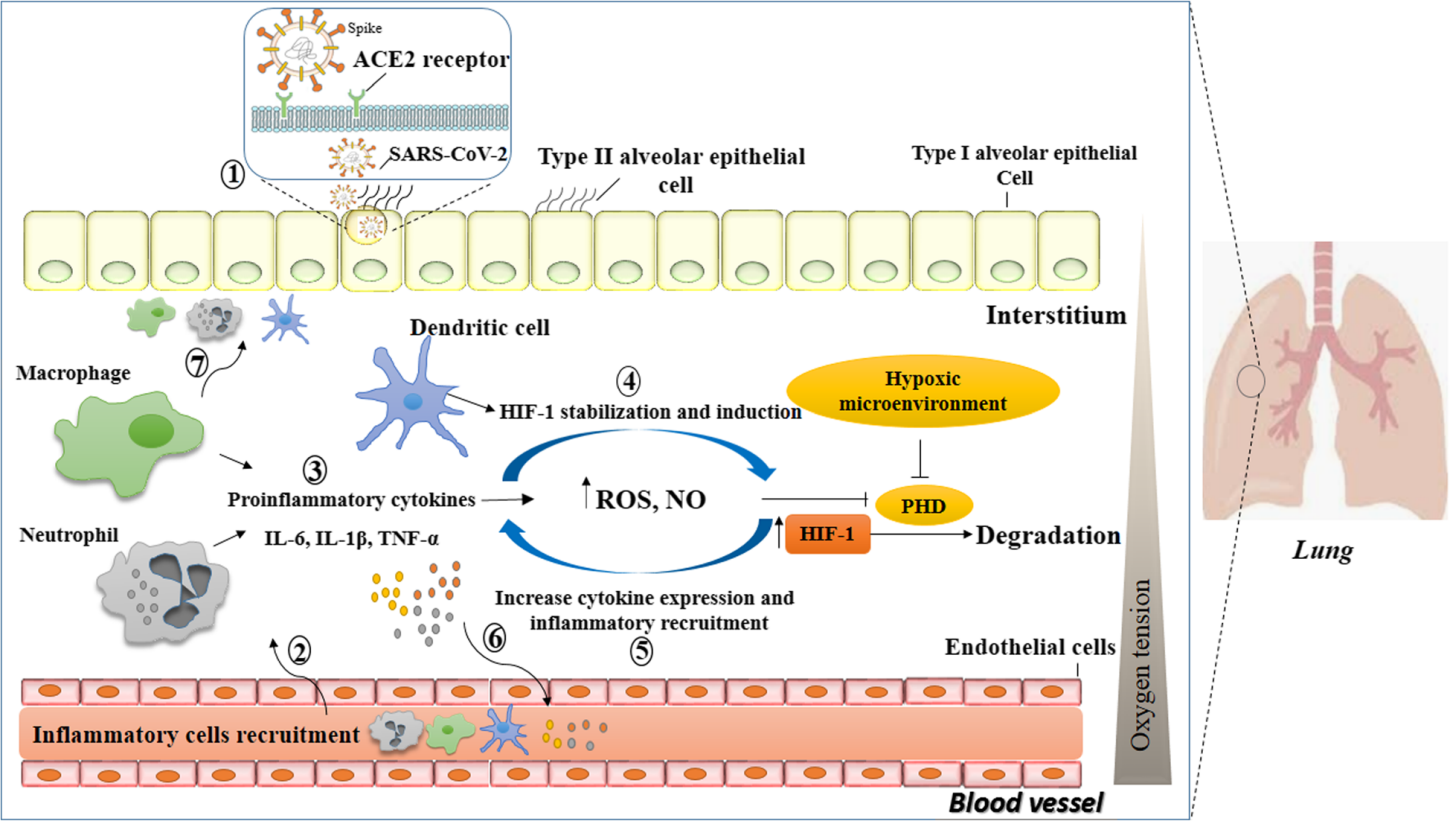
Schematic illustration of SARS-CoV-2 infection and the role of HIF-1α on SARS-CoV-2 pathogenesis. (1) SARS-CoV-2 is attached to the ACE2 receptor on the cell surface of the type II alveolar epithelial cells. After the virus entrance into the cell, its proliferation and progression leads to cell damage and (2) recruitment of the inflammatory cells into the interstitium space. Subsequently (3, 4) HIF-1α induction in the hypoxic and inflammatory condition not only stabilizes inflammatory cells including macrophages, neutrophils and DCs but also (5) induces cytokine production by this cells and cytokine storm. (6) The increasing amount of cytokine and chemokine production result in (7) increasing of the inflammatory cells recruitment into the inflammatory and infection site

As previously mentioned, SARS-CoV-2 pathogenesis is started by specific recognition of ACE2 on the surface of the ACE2 positive cells including the alveolar type II cells (AT2) and capillary endothelium. Therefore, these cells are infected by the virus followed by inflammation and hypoxia which induces HIF-1α transcriptional activity [66]. In severe cases of COVID-19, HIF-1α activation can lead to cytokine storm by activation and stabilization of immune cells including macrophages and neutrophils causing the production of high amounts of inflammatory cytokines by these cells, vascular leakage (by up-regulation of the VEGF) and destruction of the alveolar-interstitial-endothelial epithelial complex barriers (Fig. 2).

Furthermore, the possible effect of the HIF-1α on the COVID-19 pathogenesis and ARDS symptoms in patients can be explained through its relevant role in other component of the immune system including the complement system [67]. According to previous studies, C3a and also C5a fractions of complement system have important roles in the pathogenesis of the infection related-lung injury. So that, high level of C3a in the patient’s serum can predict the ARDS, and both C3a and C5a can enhance the endothelial permeability as well as macrophages and neutrophils activity and increase the cytokine production [68–70].

All together, these processes can lead to ARDS and patient^’^s death with SRS-CoV-2 infection. Thus, it is not surprising that inhibition of this transcription factor activity or blocking of its related signaling pathway can result in intense reduction of COVID-19 symptoms and patients mortality.

Consequently, in this case different pharmacological strategies for HIF-1α inhibition have been used at different levels of its expression and activity (Table.1) including those strategies preventing HIF-1α mRNA expression, translation and its transition from cytoplasm into nucleus as well as its transcriptional activity [71].

**Table 1:** List of HIF-1α inhibitors [71]

HIF-1α inhibitors	Target	Mechanism of inhibition	Pharmacological use
Aminoflavone, EZN-2698	HIF-1α mRNA	HIF-1α mRNA expression	*Therapeutic* agents for breast cancer and advanced renal cell carcinoma (RCC)
Topotecan, EZN-2208, SN38, Irinotecan	Topoisomerase I	HIF-1α translation	They are used to treat cancer of the ovaries and lymphoma
Temsirolimus, Everolimus, Sirolimus	mTOR		*They are used in breast cancer metastatic* renal cell carcinoma treatment
LY294002, Wortmannin	PI3K		They are used to inhibit the solid tumors progression including breast cancer
Digoxin, Ouabain Proscillaridin	HIF-1α protein		Therapeutic agents for congestive heart failure
2ME2, ENDM-1198, ENMD-1200, ENMD-1237	Microtubules (disruption)		Antimetastatic agents
Radicicol, KF58333 SCH66336, Apigenin Hsp90 GA, 17-AAG, 17AG, 17-DMAG	Hsp90	HIF-1α stabilization	Anti-inflammatory and anti-cancer agents
LW6	HDAC/VHL		Treatment and prevention of cancer
Acriflavin	PAS-B	HIF-1α dimerization	Antiseptic & Anti-Bacterial agent
Echinomycin	5ˊ-CGTG-3ˊ	HIF-1/DNA binding	Antibiotic agent
Doxorubicin, Danuorubicin	HRE		Anticancer agents
Chetomin	CH1domain of p300	HIF-1 transcriptional activity	Antimicrobial agents with enhancing radiotherapy effect
Bortezomib	C-TAD of HIF-1α and Asn803 of FIH		Anti-cancer agent for the treatment of relapsed and refractory multiple myeloma
YC −1, PX-478	HIF-1protein/FIH	HIF-1α at multiple levels	Anti-cancer agents

## Conclusion

Innate immunity is the first line of body defense against pathogens including viral infection. Innate immunity response to pathogens is rely on some important immune cells such as phagocytes (neutrophils and macrophages) which their hyper activity can lead to production of high amount of inflammatory cytokines and creation of phenomenon called “cytokine storm” in the site of infection. SARS-CoV-2, the new known respiratory virus, has been shown to result in high inflammatory response and some other symptoms such as ARDS in the severe cases of the disease and finally patient’s death. Given the accumulating data, HIF-1α, a crucial factor in response to the hypoxia microenvironment in the site of inflammation, acts as a ‘master regulator’ in the phagocytes. So that, it can increase the improper inflammatory responses by increasing this immune cells survival through their metabolism regulation as well as their recruitment into the inflammation site via up-regulation of the angiogenesis factor such as VEGF and following vascular permeability. Therefore, HIF-1α inhibition through pharmacological strategies might provide a new approach to aid the treatment of patients affected with COVID-19. Furthermore, in addition to the possible immune modulatory effect of HIF-1α, this transcription factor has positive impact on autophagy process. So, because of autophagy recruitment of SARS-CoV-2 in host cells to increase its proliferation and progression, HIF-1α inhibition activity is the other way to suppress viral infection. In spite of these useful effects of HIF-1α inhibition on COVID-19 symptoms, there is a challenge in this case returning to SARS-CoV-2 infection of endothelial and epithelial cells with ACE2 receptor. According to the previous studies, HIF-1α up-regulation decreases the presence of this receptor on the cell surfaces. However, due to important role of renin–angiotensin–aldosterone system and ACE2 in cardiovascular system, it is unknown whether HIF-1α inhibition can be effective strategy in COVID-19 patients, especially in those with cardiovascular diseases as highly prevalent cases among SARS-CoV2 infected patients.

## Data Availability

All data generated or analyzed during this review are included in published articles.

## Abbreviations

ACE2: Angiotensin-converting enzyme 2
ALI: Acute lung injury
ARDS: Acute respiratory distress syndrome
AT2: alveolar type II cells
*bHLH*: basic helix-loop-helix
BP: binding protein
COPD: chronic obstructive pulmonary disease
CoV: Coronaviruses
COVID-19: Coronavirus disease 2019
CREB: cAMP-response element binding protein
C-TAD: C-terminal TAD
DCs: Dendritic cells
2-DG: 2-deoxyglucose
ds: Double-stranded
EBV: Epstein- Barr virus
FIH-1: Factor inhibiting HIF-1
G-CSF: Granulocyte-colony stimulating factor
GLUTs: Glucose transferases
*HBV*: Hepatitis B virus
*HBx*: *HBV*-encoded X protein
HCV: Hepatitis C virus
HIF-1α: Hypoxia inducible factor-1α
HREs: hypoxia-response elements
IL-6: Interleukin 6
IFNI: *Interferon I*
INOS: Inducible nitric oxide synthase
IP-10: Interferon γ–induced protein-10
IPF: Idiopathic *pulmonary fibrosis*
*IRF3*: *Interferon Regulatory Factor 3*
*JAK/STAT*: Janus kinase/signal transducers and activators of transcription
*LMP1*: Latent membrane protein
MAVS: mitochondrial antiviral-signalling protein
MERS: *Middle east respiratory syndrome*
MCP-1: Monocyte chemoattractant protein-1
MIP-1A: Macrophage inflammatory proteins-1A
NF- κB: Nuclear factor-κB
NLRs: NOD-like receptors
NSCLC: Non-small cell lung cancer
N-TAD: N- terminal TAD
ODDD: Oxygen dependent degradation domain
OXPHOS: Oxidative phosphorylation
PAMPs: Pathogen-associated
molecular: patterns
PAS: Per-Arnt-Sim
PDK1: Pyruvate Dehydrogenase Kinase 1
PHDs: Prolyl-hydroxylase domain proteins
PKM2: Pyruvate kinase isozymes M2
PKR: Protein kinase R
PRRs: Pattern recognition receptors
RIG-I: Retinoic acid inducible gene-I
RLRs: RIG-I-like receptors
SARS: Severe acute respiratory syndrome SS Single-stranded
STAT3: Signal transducer and activator of transcription 3
TAD: Transactivation domains
TCA: Tricarboxylic acid cycle
TLRs: Toll like receptors
TNF-α: Tumor necrosis factor alpha
TRAF3: Tumor necrosis factor receptor (TNFR)-associated factor 6
VEGF: Vascular endothelial growth factor
